# Sex-biased gene expression in nutrient-sensing pathways

**DOI:** 10.1098/rspb.2022.2086

**Published:** 2023-03-08

**Authors:** Suzanne Bennett-Keki, Emily K. Fowler, Leighton Folkes, Simon Moxon, Tracey Chapman

**Affiliations:** School of Biological Sciences, University of East Anglia, Norwich Research Park, Norwich NR4 7TJ, UK

**Keywords:** diet, lifespan, nutrient-sensing, fruitfly

## Abstract

Differences in lifespan between males and females are found across many taxa and may be determined, at least in part, by differential responses to diet. Here we tested the hypothesis that the higher dietary sensitivity of female lifespan is mediated by higher and more dynamic expression in nutrient-sensing pathways in females. We first reanalysed existing RNA-seq data, focusing on 17 nutrient-sensing genes with reported lifespan effects. This revealed, consistent with the hypothesis, a dominant pattern of female-biased gene expression, and among sex-biased genes there tended to be a loss of female-bias after mating. We then tested directly the expression of these 17 nutrient-sensing genes in wild-type third instar larvae, once-mated 5- and 16-day-old adults. This confirmed sex-biased gene expression and showed that it was generally absent in larvae, but frequent and stable in adults. Overall, the findings suggest a proximate explanation for the sensitivity of female lifespan to dietary manipulations. We suggest that the contrasting selective pressures to which males and females are subject create differing nutritional demands and requirements, resulting in sex differences in lifespan. This underscores the potential importance of the health impacts of sex-specific dietary responses.

## 1. Introduction

Sex differences in lifespan and in disease incidence are pervasive, but whether there are unifying reasons for them remains unclear. Such differences are influenced by the fundamental selective forces that result in sexual dimorphism across all aspects of organismal physiology [1]. Three main hypotheses have been proposed to explain ultimate causes for sex differences in lifespan. The first arises from the ‘unguarded’ nature of the maternally inherited X chromosome in males, meaning that X-linked genes with deleterious impacts on male lifespan and fitness may be expressed [2–5]. A related explanation concerns ‘toxic Y’ effects on lifespan [6]. A second hypothesis stems from the asymmetric, maternal, inheritance of mitochondria (mt). Hence mutations carried by mtDNA that are detrimental to males are less strongly selected against, than those with adverse effects on females (i.e. the ‘mother's curse’ [7–11]). A third hypothesis stems from sex-specific selection over many aspects of male and female life history [12–17]. A key role for sex-specific selection comes from observations of associations of lifespan with mating systems [15]. Hence sexual dimorphism in lifespan and ageing may result from sex-specific trade-offs between longevity and reproduction [12,18–21].

In terms of the directionality of lifespan differences, females are generally assumed to live longer in natural contexts. However, there is considerable variation across and within species in terms of which sex generally lives longest [1,17,20]. For example, in humans, cats, rats, pilot whales and many species of monkeys, females generally outlive males, whereas in dogs and some bats lifespans are very similar or males live longer. Counter-examples to the major hypotheses described above are also known. Thus, whether there are general unifying principles determining the directionality of sex differences in lifespan is unclear [1].

Whatever the ultimate explanation, it is clear that sex differences in lifespan persist and are associated with distinct profiles of health and disease. For example, women have been reported to live longer than men since UK records began in 1841 and deaths from heart disease, cancer and diabetes mellitus are more common in men, while women have a higher incidence of death due to cerebrovascular disease, osteoporosis, autoimmune disorders and Alzheimer's disease [22,23]. Length of life has also long been linked to dietary intake. For example, experiments in rats first showed that dietary restriction led to an increase of approximately 30% in length of life [24]. Since then, the effect of dietary restriction on longevity has been described in many taxa [25–31]. The quality and quantity of specific nutrients have pervasive and robust effects on lifespan and reproductive success [32,33], with the balance between protein and carbohydrate being critical [34–37]. Furthermore, the nutritional requirements of each sex can differ. For example, in *D. melanogaster*, protein is required for production of eggs and higher protein intake occurs in mated over virgin females [38,39]. A male's reproductive success can be increased via the intake of carbohydrates, to produce energy for finding and attracting mates [35,40]. Nutritional inputs of macronutrient ratio, such as proteins and carbohydrates, can alter sex-specific phenotypes such as egg production and laying, male calling behaviour and digestive efficiency [41,42]. The ingestion of the monosaccharide d-galactose, present in milk, fruit and vegetables, is reported to induce distinct behavioural outcomes in male and female mice, in a dose-dependent manner, and with opposing effects on key senescence traits [43]. Overall, the emerging picture is that each sex has nutritional requirements that have sex-specific effects on lifespan and health.

**Figure 1.**
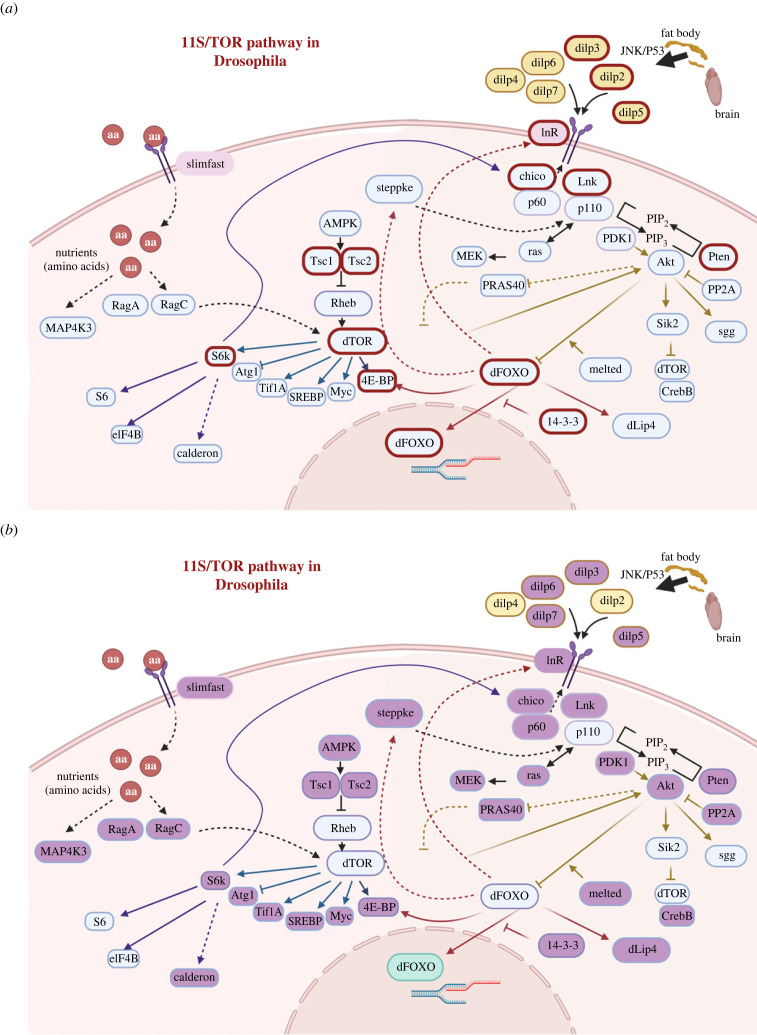
IIS/TOR signalling network in *Drosophila* illustrating IIS/TOR genes with (*a*) previously reported effects on lifespan determination and (*b*) significant sex-biased gene expression as determined in this study. The IIS/TOR pathway is shown, with extracellular, intracellular and nuclear components. Arrows indicate activation steps and bar-ended lines indicate inhibitory interactions. Broken lines indicate indirect or potential interactions. (*a*) IIS/TOR genes previously reported to play a role in lifespan determination in *D. melanogaster* (electronic supplementary material, table S1) are bordered in red. (*b*) highlighted in purple is a summary of the IIS/TOR genes from this study that have the potential to show significantly sex-biased gene expression from RNA-seq data (in either body part in virgin or mated adult flies), or from qRT-PCR (in 5 or 16-day-old once mated adult males or females). The nutrient-sensing pathway shown is adapted from Partridge *et al*. [47] and Teleman [48]. Figures created under publication licence using Biorender.

Evidence showing that manipulations of diet have robust, and potentially sex-specific effects, on longevity has prompted much interest in whether these effects are mediated by nutrient-sensing pathways. A huge body of research now shows that the activity of the insulin/insulin like growth factor (IIS) and target of rapamycin (TOR) pathway is associated with lifespan determination across a huge range of animal species [44]. IIS and TOR pathways are highly conserved [45,46] and many components of them have been shown to affect lifespan directly (figure 1, electronic supplementary material, table S1; [47–49]). For example, in *Drosophila melanogaster*, the level of expression of insulin-like peptide genes (*dilp1* and *dilp2*) [50] secreted from insulin producing cells in the brain can interact to regulate ageing [51]. Dietary restriction-mediated lifespan extension is also associated with *dilp5*, and over-expression of *dilp6* in the adult fat body leads to extended lifespan [52]. Loss of the intracellular substrate encoded by *chico* also extends lifespan in *Drosophila* [53] as does loss of function of the IIS regulator *Lnk* [54] and activation of *4E-BP* [55]. Related findings are also found in other model systems, for example, suppression of insulin-induced Akt signalling in *C. elegans* increases longevity [56], elevated *Tsc1* expression increases longevity in female mice [57], S6k1 affects both health and lifespan in mice [58] and FOXO expression has direct effects on longevity in several species [59,60].

Whether there is differential activity in nutrient-sensing pathways associated with sex differences in lifespan is the key hypothesis we test here. Our rationale is the emerging finding that direct manipulations of diet or IIS/TOR genes as described above often result in very different outcomes in each sex. For example, in *D. melanogaster*, nematodes and mice the benefits of dietary restriction result in larger effects on lifespan in females [17,61]. Similarly, perturbations to the IIS and TOR pathways can also result in sex-specific effects on lifespan [53,62].

In this study, we examined whether there was any evidence that the divergent responses of health and lifespan of each sex to differing diets, and specifically the high nutritional sensitivity of females in particular, is underpinned by sex differences in the expression of IIS/TOR network genes with reported effects of lifespan. The specific hypothesis was that nutrient-sensing genes with lifespan effects would show more activation, and more dynamic expression over time, in females than males. We tested for sex-biased expression in 17 nutrient-sensing genes in *Drosophila melanogaster* that have reported effects on lifespan [47] (electronic supplementary material, table S1; figure 1). We first used a published RNA-sequencing dataset [63] to undertake a new analysis to test for sex-biased expression of nutrient-sensing gene expression in both virgin and mated males and females (in two different body parts, the Head + Thorax and Abdomen). We then undertook direct tests for sex-biased expression of the same 17 nutrient-sensing genes in larvae and once mated, young and older males and females (whole individuals). Our predictions were that the differential sensitivity of female lifespan to dietary manipulations would be associated with significant female-biased gene expression in IIS / TOR genes, and secondly, that these nutrient-sensing genes would be more sensitive to reproductive state in females than males.

## 2. Methods

### (a) Analysis of nutrient-sensing genes extracted from whole transcriptome RNA-sequencing of virgin and mated males and females (in head + thorax and abdomen body part samples)

We first reanalysed the mRNA-seq dataset of Fowler *et al*. [63]. In that original study, differences in the expression of mRNAs in the head/thorax (HT) and abdomen (AB) body parts of virgin versus mated males or females were reported. In this study, we tested specifically for sex-biased gene expression in virgin versus mated samples (by comparing the expression level of genes in males versus females directly). Raw sequencing reads (accession PRJNA521155) were downloaded from the Sequence Read Archive (SRA) [64] in FASTQ format. Reads were trimmed to remove both poor-quality calls and adapters using Trim Galore! (v. 0.3.4) [65] with default settings. Quality control checks were carried out using FastQC (v. 0.11.8) [66] with default settings, both before and after adapter and read quality trimming. Differential gene expression analysis was performed using the Berkeley *Drosophila* Genome Project (BDGP6.28) genome and gene annotation in GTF format downloaded from Ensembl (release 90) [67]. Trimmed reads were aligned to the genome using HISAT2 (version 2.1.0) [68] with single-end and unstranded settings (all other parameters set as default). Quality control of mapping data was performed on the resulting BAM files using QualiMap RNA-Seq QC (v. 2.2.2) [69] with default settings. Gene counts were extracted from the BAM file for each sample using the GTF annotations and htseq-count (v. 0.9.1) [70] run with unstranded settings (all other parameters default). Differential expression analysis was performed using default settings in DESeq2 (v. 1.22.1) [71] via the Galaxy platform (https://usegalaxy.eu/) [72]. We normalized and analysed the mRNA-seq data to focus specifically on comparisons between the sexes - to test for sex differences in mRNA expression and detect the extent to which genes show higher or lower gene expression in males versus females. We then extracted from that dataset the IIS/TOR network genes (figure 1*a*) and compared their patterns of sex-biased gene expression.

### (b) Direct quantification of nutrient-sensing gene expression in male and female larvae, and 5- and 16-day-old adults (whole larvae and adult samples)

To complement the above analysis, we undertook independent, direct tests for sex-biased patterns of expression in nutrient-sensing genes, by using quantitative RT-PCR. Wild-type *D. melanogaster* flies from a large laboratory population originally collected in the 1970s in Dahomey (Benin) were used. Experimental flies were obtained following 2 generations of standard rearing, to minimize parental carry over effects. Eggs were collected from purple grape juice media Petri dishes (1.342 l water, 61 g agar, 0.73 l red grape juice, 51 ml Nipagin 10% w/v) that had been placed for 2–3 h in three stock cages. Plates were then removed, divided into 4 and each quarter placed in a 1/3 pint glass bottle containing 70 ml SYA medium (100 g brewer's yeast powder, 50 g sugar, 15 g agar, 30 ml Nipagin (10% w/v solution) and 3 ml propionic acid, per litre of medium). This gave 4 bottles for each of 3 replicate stock cages which were then incubated (25°C, 50% humidity, 12 : 12 hour light:dark cycle). The emerging flies were placed in small egg-laying cages over purple grape juice media Petri dishes for 3–4 h. Eggs laid were transferred into glass vials (75 mm × 24 mm) each containing 8 ml SYA medium, at a density of 50 per vial (3 vials for each biological replicate). Ten larvae were sampled at the wandering instar 3 stage (day 5 from vial set up) at random from each vial for all replicates and placed directly in 1.5 ml tubes in a −80°C freezer. The day on which there was peak adult emergence was designated ‘day 0’. The experimental flies emerging on this day were allowed to mate for 24 h and then sex separated and stored in single sex groups of 5 in vials and transferred to fresh food every 2–3 days. On Day 5 and Day 16 after eclosion the adults were frozen at the same time of day (120 min after lights on). Flies were briefly anaesthetized using CO_2_, transferred into 1.5 ml tubes and then immediately placed into an −80°C freezer to await RNA extraction. We reasoned that this method of transfer would more easily standardize handling across samples than the alternatives of blowing or shaking flies into the tubes. All methods of transfer to the freezer have potential effects on gene expression. However, the gene patterns we describe are over and above any transfer effects and not expected to be confounded by them.

#### (i) RNA extraction

We extracted RNA from whole individuals of single larvae, and from groups of 5 adult flies from each sex for each sample day (day 5 and day 16). There were three biological replicates of each sample and RNA was extracted from whole larvae and adults. Tissues were disrupted by grinding using an electric micro pestle, and total RNA extracted (miRvana kit, Ambion, AM1561) according to the kit protocol (with adjustment of the initial lysis solution into 50 µl followed by 150 µl, to ensure proper grinding of the material). RNA was eluted in RNA storage solution (1 mM sodium citrate, pH 6.4 ± 0.2, Ambion). Samples were DNase treated (Ambion Turbo DNA-free kit, AM1907). RNA was assessed for quantity and quality using a NanoDrop 8000 spectrophotometer. cDNA was synthesized using the Revertaid RT kit for reverse transcriptase (*Thermo Scientific* K1621) by following the kit protocol, and stored at −20°C.

#### (ii) Sex identification of larvae

We used a molecular method. *βtubulin85D* (FBgn0003889) is reported to be expressed specifically in testes [73] predicting a dimorphic pattern of *βtub85D* expression, high in males and low in female larvae. We first verified this by sexing a subset of larvae by eye on the basis of the larger testes versus ovary imaginal discs. We then quantified the level of *βtub85D* expression in the same larvae, normalized to reference genes *elF1A* (FBgn0026250) and *αTub84B* (FBgn0003884) (electronic supplementary material, table S2). As expected, larvae sexed as males had high *βtub85D* expression and larvae sexed as females had no *βtub85D* expression above background noise. Thus we used *βtub85D* expression assays to identify the sex of experimental larvae.

#### (iii) RT-PCR

Quantitative RT-PCR was performed using a Bio-Rad CFX Connect Thermal Cycler (software CFX maestro) and iTaq universal SYBR green supermix (Bio-Rad no. 1725121). Primers were manufactured salt-free *Eurofins Genomics* provider; electronic supplementary material, table S2). Primer efficiencies were checked using a 5-fold standard curve of cDNA with a maximum input of 50 ng total cDNA, and primer concentrations that yielded efficiencies of between 90 and 110% were determined (electronic supplementary material, table S2). Relative quantities of target transcripts were normalized using 2 reference genes, *elF1A* and *αTub84B* whose expression was stable across each sex (see raw data file electronic supplementary material, table S10). To avoid intra-plate variation, all samples for stage and sex were loaded onto a single qPCR plate for each set of primers. Sufficient stock cDNA at 2 ng/µl was prepared for all target primers and for each RT-PCR run a mastermix of primers and SYBR iTaq (62 µl forward primer, 62 µl reverse primer, 620 µl SYBR iTaq and 186 µl molecular grade water) prepared before aliquoting into each well (15 µl mastermix and 5 µl cDNA) of a 96-well plate (Bio-Rad MLL-9601). Plates were sealed with an adhesive film (Bio-Rad MSB-1001). The mean Ct value for both reference gene expression was used to normalize cDNA for each sample by subtracting it from the mean target gene expression *C*_t_ value. Relative gene expression was then calculated using the 2^−Δ*C*t^ method [74]. One male sample (male, day 16, replicate 3) was removed from the dataset as it was identified as a statistically significant outlier using the Grubbs Test. A two-way ANOVA was then used to test for differences in relative expression of nutrient-sensing genes (R v. 4.1.1 [75]). Sex and life stage were designated as factors in the model and we also tested for interactions between them. Post hoc Tukey tests were subsequently used to detect between which life stages any differences in gene expression had occurred.

## 3. Results

### (a) Sex-biased expression in nutrient-sensing genes

#### (i) General patterns of sex-biased gene expression—RNA-sequencing data

Expression data for the genes in figure 1*a* were obtained from the analysis of the RNA sequencing dataset previously provided by Fowler *et al*. [63] (electronic supplementary material, tables S3 and S4). Genes were called as significantly differentially expressed if they passed the stringent threshold of showing a greater than log 2 fold change (±2log2FC) and an adjusted *p*-value of < 0.05 from the DEseq2 analysis (with the adjusted *p*-value accounting for the effects of multiple comparisons). Significant sex-biased gene expression (a significantly greater level of gene expression in one sex than the other, in either direction) was detected in the majority of genes involved in the IIS/TOR network (figure 1*b*). Out of the 44 IIS/TOR genes examined, 35 showed evidence for significant sex bias in gene expression in at least one body part in virgin or mated flies. Across both body parts and in virgins and mated flies, consistently more sex biased nutrient-sensing genes showed female biased (FB) in comparison to male biased (MB) expression (electronic supplementary material, tables S3 and S4). Among the sex biased genes in the whole transcriptome data, more genes showed FB expression in the head + thorax, and more of them MB in the abdomen (electronic supplementary material, table S3). Thus, more nutrient-sensing genes showed a pattern of FB expression in the abdomens than expected in comparison to whole transcriptome data (electronic supplementary material, table S3), and nutrient-sensing genes showed a pattern of FB expression overall.

#### (ii) General patterns of sex-biased gene expression—qRT-PCR data

**Figure 2.**
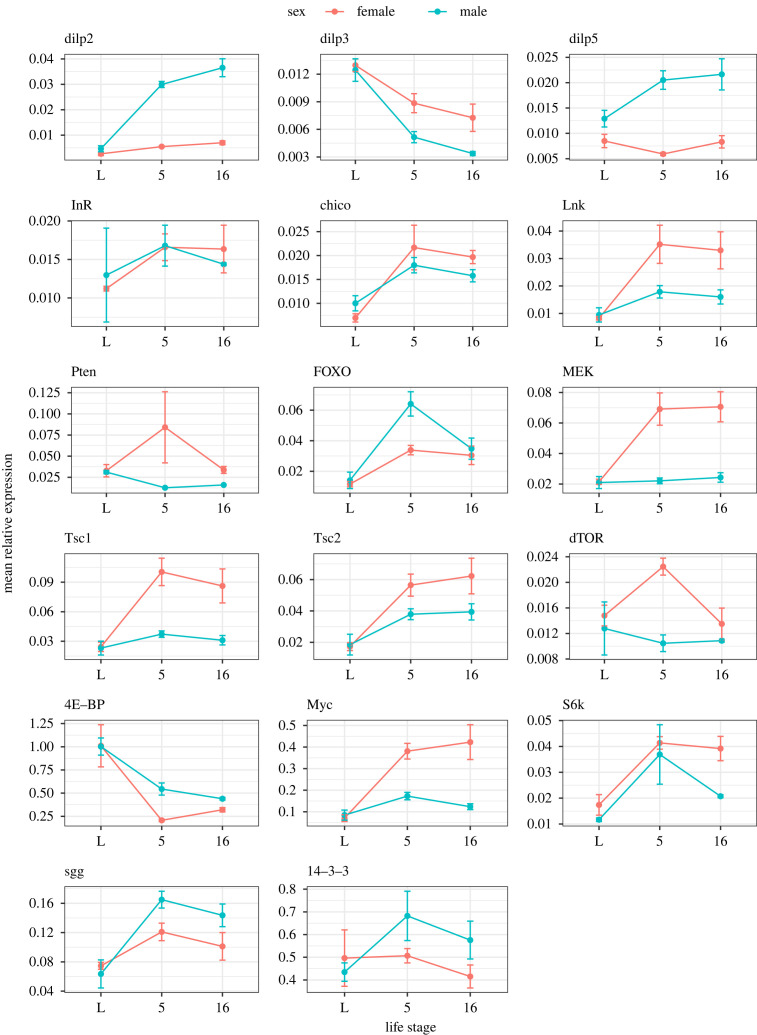
Relative expression levels in IIS/TOR nutrient-sensing pathway genes at three different life stages, as determined by qRT-PCR. Shown on the *x*-axis are the data for larvae (L), young adults day 5 (5) and old adults day 16 (16), for males and females. Normalized, relative gene expression is shown on the *y*-axis calculated using the 2^−Δ*C*t^ method. Red = females; green = males.

Consistent with the above, direct tests for differences in expression of nutrient-sensing genes by using a 2 way ANOVA followed by *post-hoc* testing in wild-type males and females also revealed that significant sex bias was common (10 out of 17 genes tested showed significant effects of sex × life stage or of sex alone, with an additional locus (*Pten*) showing non-significant sex bias (*p* < 0.1) (figure 2; table 1; electronic supplementary material, tables S5 and S6). For the nutrient-sensing loci in which a significant difference in stage was identified, *post hoc* testing confirmed that the difference occurred between the larval and adult stages, and almost never between day 5 and day 16 adults. In general, larval gene expression was significantly lower than in adults, except for *4EBP* and *dilp3*. For many genes there appeared to be a trend for differences in expression of day 5 to day 16 adults, but this was significant only for *FOXO* in which there was significantly higher expression at day 5 in males. Nutrient-sensing loci showed significant sex-biased expression only in adults. Loci showing a significant main effect of sex (*dilp3*, *Lnk*, *Tsc2* and *dTOR*) had higher expression in females (figure 2; table 1; electronic supplementary material, tables S5 and S6). Similar expression patterns were observed for *dilp2* and *dilp5*, with male expression being significantly higher in adult males compared to females. A similar but sex-reversed pattern was observed for *Dm*, *MEK* and *Tsc1*, with expression being significantly higher in adult females. *FOXO* showed significantly higher expression in young male adults. *dTOR* appeared to show higher expression in young females but this effect was not significant. Overall, there was consistency in the findings from the high and low throughput methods of quantifying sex-biased gene expression. The qPCR data confirmed that significant sex-biased expression of nutrient-sensing genes in adults is typical, and both datasets confirmed a pattern of female-biased expression in nutrient-sensing genes (electronic supplementary material, table S6).

**Table 1.** Summary of IIS/TOR nutrient-sensing genes identified as showing significant differences in gene expression across sexes or life stages, from the qRT-PCR data. Shown are the summary results for the expression of 17 nutrient-sensing loci analysed by qRT-PCR in electronic supplementary material, table S4. Ticks indicate if there was a significant main effect of sex or stage, or their interaction (sex × stage) at *p* < 0.05.

gene	Fbgn number	sex × stage	sex	stage
*dilp2*	FBgn0036046	✓		
*dilp3*	FBgn0044050		✓	✓
*dilp5*	FBgn0044048	✓		
*InR*	FBgn0283499			
*chico*	FBgn0024248			✓
*Lnk*	FBgn0028717		✓	✓
*Pten*	FBgn0026379			
*FOXO*	FBgn0038197	✓		
*MEK*	FBgn0010269	✓		
*Tsc1*	FBgn0026317	✓		
*Tsc2*	FBgn0005198		✓	✓
*dTOR*	FBgn0021796		✓	
*S6k*	FBgn0283472			✓
*Myc*	FBgn0262656	✓		
*4E-BP*	FBgn0261560			✓
*sgg*	FBgn0003371			✓
*14-3-3*	FBgn0004907			

#### (iii) Sex-biased gene expression in different parts of the nutrient signalling pathway

To check for patterns of sex-biased gene expression across the nutrient signalling pathway in more detail, we divided it into upstream to downstream sections (electronic supplementary material, figure S1a–f; table S8) and examined the direction of sex-bias (as indicated by the analysis derived from the RNA-seq data). This analysis again highlighted the general dominance of FB gene expression, but with no particular focus of either FB or MB genes in any specific part of the nutrient-sensing pathway. The qRT-PCR data were consistent with these findings (electronic supplementary material, table S7) and showed that throughout the nutrient-sensing pathway there were nutrient-sensing loci showing expression differences due to sex, life stage or their interaction (figure 2; table 1; electronic supplementary material, tables S5 and S6).

### (b) Changes in sex-biased expression in nutrient-sensing genes upon mating

Analysis of the RNA-seq data allowed us to probe whether sex-biased gene expression patterns changed upon mating. In general, mating did not result in a change in the dominant pattern of FB expression of nutrient-sensing genes in the abdomen (table 2). 23 nutrient-sensing genes showed significant FB expression before and 22 after mating. 5 genes showed significant MB in virgin abdomens and 6 in mated flies. Of those genes that did change pattern upon mating in the abdomen, *Pdk1* was significantly FB before, but lost this pattern after mating, while *SREBP* expression showed no sex bias before and became MB after mating. By contrast, sex-biased patterns of expression in nutrient-sensing genes in the head + thorax varied significantly before and after mating. In virgins there were 9 FB and 5 MB genes, but only 4 FB genes in mated flies. Both MB and FB genes in the head + thorax of virgins lost their sex bias in mated flies. 4 genes showing no sex bias in virgin head + thorax gained a FB pattern after mating (table 2; electronic supplementary material, table S9). The dominant pattern in the head + thorax was the loss of sex bias upon initiation of reproduction: 10/4 genes lost/gained FB expression and 5/1 genes lost/gained MB expression. In the head + thorax of males, expression was reduced in 16 of the 18 nutrient-sensing genes after mating, while none showed an increase of expression following mating. In females, 9 genes showed lower expression after mating, with 6 having increased expression. When a non sex-biased gene became FB following mating, this was typically driven by a reduction in male expression, in some cases accompanied by an increase in female expression. Changes from FB to NS arose from either a reduction in gene expression in females or a reduction in both males and females. The genes showing a change in pattern from MB to NS resulted from either a reduction in gene expression in males, or both a reduction in male and increase in females. In the two genes showing change in sex bias in the abdomen after mating (PDK1 lost FB to become NS, while SREBP changed from NS to MB), an increase in expression was seen in both sexes, however, the direction in both instances was with greater expression in males (table 2; electronic supplementary material, table S9).

**Table 2.** Summary of IIS/TOR nutrient-sensing genes showing a significant switch to or from sex-biased gene expression upon mating, from the RNA-seq data (±2log2FC and an adjusted *p*-value of less than 0.05). FB = female-biased, MB = male-biased, NS = no sex bias. Shown are nutrient-sensing genes for which there was evidence of a significant change in the pattern of sex-biased expression upon mating, from the RNA-sequencing data.

gene	FBgn number	body part	sex-biased expression	
			virgin	mated
*dilp3*	FBgn0044050	HT	NS	FB
*dilp 5*	Fgbn0044048	HT	FB	NS
*dilp 6*	FBgn0044047	HT	NS	FB
*chico*	Fgbn0024248	HT	MB	NS
*Lnk*	Fgbn0028717	HT	FB	NS
*steppke*	Fgbn0086779	HT	MB	NS
*Ras*	Fgbn0003204	HT	FB	NS
*PDK1*	Fgbn0020386	AB	FB	NS
*Pten*	Fgbn0026379	HT	MB	NS
*AMPK*	Fgbn0023169	HT	FB	NS
*Sik2*	Fbgn0025625	HT	FB	NS
*4E-BP*	Fgbn0261560	HT	FB	NS
*14-3-3*	Fgbn0004907	HT	MB	NS
*S6k*	Fbgn0283472	HT	NS	FB
*TIF1A*	Fgbn0032988	HT	MB	NS
*SREBP*	Fgbn0261283	HT	FB	NS
*SREBP*	Fgbn0261283	AB	NS	MB
*Myc*	Fgbn0262656	HT	NS	FB
*calderon*	Fgbn0086365	HT	FB	NS
*MEK*	FBgn0010269	HT	FB	NS

### (c) Changes in sex-biased expression in nutrient-sensing genes across different life stages

The qRT-PCR analysis of expression changes in nutrient-sensing genes across life stages showed sex differences in expression were not established until adulthood. Patterns of sex bias in either direction were also consistent across young and older adults (i.e. did not cross over; figure 2**)**. 9 genes showed significant FB and 4 MB in adults, with none showing sex bias in larvae. Most nutrient-sensing genes also had higher expression in adults than in larvae.

## 4. Discussion

Overall, the findings were generally consistent with the hypothesis that nutrient-sensing genes with lifespan effects would show more activation, and more dynamic expression over time, in females than males. In line with our first prediction, there was an overall pattern of female-biased gene expression in lifespan-influencing IIS/TOR genes. Following the second, these nutrient-sensing genes also appeared to be more sensitive to reproductive state in females than males, with a general loss of female-biased expression upon mating.

That many of the IIS/TOR pathway genes tested showed significant sex bias is not surprising, given that there is sex biased expression in the majority of protein-coding genes. However, there was no consistent directionality in the sex bias of gene expression across the whole comparator transcriptome in the RNA-seq data analysed, whereas among nutrient-sensing genes, there were more FB than MB genes found across all body parts and in virgin and mated flies. This pattern was confirmed directly by using qRT-PCR. The pattern of sex-biased expression of nutrient-sensing genes was also observed to change significantly upon the transition to the mated state and this pattern differed across body parts, with the patterns of sex bias remaining stable in abdomens but being much more dynamic in the head + thorax. When changes in the patterns of sex-biased expression occurred, it was generally due to the loss of FB expression. Results from RNA-sequencing and qRT-PCR showed that no particular up- or downstream part of the nutrient-sensing pathway was more or less dominated by FB or MB expression. Nutrient-sensing genes were never sex-biased in expression during the larval stage, but developed patterns of sex bias in adulthood, which generally remained consistent. The changing dynamics of sex-biased gene expression across life stages and with mating status show that elevated FB expression in nutrient-sensing genes is not simply a consequence of females generally having larger body sizes than males. Overall, these results suggest that the greater sensitivity of female lifespan to diet could be associated with greater activation in their nutrient-sensing genes. We suggest that the consistent loss of FB patterns of gene expression could underlie the generally greater survival costs of reproduction in females than males.

The genes chosen for study were the nutrient-sensing genes with reported effects on lifespan (electronic supplementary material, table S1). These were initially identified as affecting lifespan mostly via tests with mutant strains, direct assays or by genetic interactions (electronic supplementary material, table S1). Those findings highlight the central importance of these genes in determining length of life. However, studies of the relationships of segregating genetic variation in nutrient genes and longevity in natural populations remain scarce. Studies of the functional differentiation between different *dilp* nutrient-sensing genes across *Drosophila* suggests that this pattern may have been selected because it confers fitness benefits [50]. However, the significance of genetic variation in nutrient-sensing genes in natural populations remains unclear, as is how any sex-specific regulatory architecture is encoded.

The results are of general relevance because nutrient-sensing genes show deep conservation and effects of these genes on lifespan are found across may different taxa [47]. This could suggest that the activation of nutrient-sensing genes is a general contributor to variation in male and female lifespan. The results also beg the question of whether other physiological processes might exhibit similarly dynamic patterns of sex-biased gene expression. Differences between the sexes and some sex-biased gene expression have been reported, for example, in energy metabolism, immunity, excretion and neurosensory pathways. We describe a few such examples in electronic supplementary material, box S1 and note that comprehensive investigations into the patterns and consequences of sex-biased gene expression in these pathways could be useful. Our results also add fundamental information to the emerging picture of how the distinct effects of diets on the life history and health of males and females might be determined. This could be of relevance to understanding the potential effectiveness of dietary interventions used to treat diseases such as type 2 diabetes [76,77]. An understanding of sex-specific changes in gene expression following the initiation of potentially costly reproductive activity might also suggest potential routes for health interventions. For example, in model systems, the drug mifepristone can block the negative effect of shortened lifespan that results in females after mating [78,79] and in humans mifepristone is used treat patients with high blood sugar [80]. Understanding the links between these effects of reproduction-induced changes in lifespan and nutrient metabolism could be useful.

### (a) Directionality in sex-biased pattern of expression in nutrient-sensing genes

The findings were generally consistent with the prediction that the greater sensitivity of female lifespan and life history to variation in diet might be manifested as an increase in the activation of nutrient-sensing gene expression. For virgin and mated flies and across both body parts (RNA-seq data) and for young and adult once mated flies (qRT-PCR data) approx. 2–4 times as many nutrient-sensing genes showed FB than MB expression. This contrasted with the comparator whole transcriptome data, which showed more FB expression in the head + thorax, but more MB in the abdomen. Thus, it seems that the nutrient-sensing pathway is characterized by a pattern of FB gene expression among sex-biased genes. This has the potential to underlie the increased sensitivity of female lifespan and life history to nutrients, assuming that higher levels of expression in such genes in females translates into phenotypic sensitivity. Many studies have examined patterns of sex-biased gene expression in terms of documenting its occurrence [81] and relevance to the field of sexual selection [82], sexual conflict [83,84] and sex chromosome linkage [85]. These studies have sought to understand how sex-specific selection can alter genome-wide patterns of sex-biased gene expression. However, to our knowledge, there are few studies so far seeking to associate effects of nutrient-sensing genes to patterns of sex-biased gene expression.

### (b) Mating alters sex-biased patterns of expression in nutrient-sensing genes

The pattern of sex bias in nutrient-sensing genes was stable before and after mating in the abdomen across all nutrient-sensing genes. By contrast, in the head + thorax the situation was more dynamic, with mating often leading to a change in sex-biased gene expression, usually the loss of FB expression in mated females. The induction of significant alterations to the pattern of sex bias in nutrient-sensing genes upon mating could potentially be associated with survival costs of mating in females. Mating and receipt of seminal fluid proteins have been shown to increase female nutrient acquisition [38], alter nutrient-sensing [39] and reduce lifespan [86–90]. It is not yet known whether/how these effects are directly linked—changes to the pattern of expression of nutrient-sensing genes in females upon mating that we describe here provide a potential bridging mechanism [79,91]. Future tests should investigate the direct links between these different facets [92].

### (c) Sex-biased expression in nutrient-sensing genes differs across different body parts

Perhaps unsurprisingly, the results suggests that sex-biased expression varied significantly across different body parts. Most work in *Drosophila* has focused on sex bias gene expression in either gonads, the brain or whole-body samples [93]. Observations of gene expression in *Drosophila* have shown that MB expression tends to be restricted to sex-specific tissues, whereas FB genes are often more broadly expressed [94]. Our analyses showed that the pattern of sex-biased gene expression in the abdomen was different to that seen in the head + thorax, with sex-biased expression in the abdomen being consistently FB before and after mating. The head + thorax was more labile. The observation of changes in the IIS/TOR gene network, particularly in females, in response to mating supports the link between longevity of females and their sensitivity to mating and nutrient-sensing.

### (d) Sex-biased expression in nutrient-sensing genes differs across the lifecourse

The direct tests of nutrient-sensing gene expression were undertaken by using qRT-PCR to determine gene expression in 17 nutrient-sensing loci reported to have effects on lifespan [47,95]. This analysis validated the dominant female-biased patterns of expression in nutrient-sensing genes observed in the RNA-sequencing and identified different patterns of gene expression across the lifecourse. The expression of nutrient-sensing genes did not differ between the sexes during development (consistent with previous evidence of lack of sexually dimorphic expression of in dilps [96]) and was generally, but not always, lower than in adults. Sex bias in the expression of nutrient-sensing genes during adulthood tended not to interact with adult age and was generally consistent in direction in young and older males and females. Whether the patterns described here differ across body parts or show interactions with mating status across age will be interesting to test in the future.

Overall, our results contribute to an increase in understanding sex-specific variation in nutritional effects and in the expression of genes in the IIS and TOR network. This offers potential in developing interventions to improve health during ageing [49] as well as giving greater understanding of mechanisms that maintain sex differences.

## Data accessibility

Raw sequencing data for this study are stored at the Sequence Read Archive (SRA) using the BioProject accession: PRJNA521155. Raw qRT-PCR data (Ct values and normalized gene expression) are appended in electronic supplementary material table S10 [97].
